# Effects of Preoperative Factors on the Learning Curves of Postlingual Cochlear Implant Recipients

**DOI:** 10.1097/AUD.0000000000001690

**Published:** 2025-05-29

**Authors:** Hendrik Christiaan Stronks, Timothy Samuel Arendsen, Mirte Veenstra, Peter-Paul Bernard Marie Boermans, Jeroen Johannes Briaire, Johan Hubertus Maria Frijns

**Affiliations:** 1 Department of Otorhinolaryngology and Head & Neck surgery, Leiden University Medical Center, Leiden, the Netherlands; 2 Leiden Institute for Brain and Cognition, Leiden University, Leiden, the Netherlands; 3 Department of Bioelectronics, Delft University of Technology, Delft, the Netherlands; 4 These authors contributed equally to this work.

**Keywords:** Auditory rehabilitation, Cochlear implant, Learning curve, Sensorineural hearing loss, Speech training

## Abstract

**Objectives:**

The substantial variability in speech perception outcomes after cochlear implantation complicates efforts to develop valid predictive models of these outcomes. Existing predictive regression models are too unreliable for clinical application, possibly because speech intelligibility (SI) after cochlear implant (CI) rehabilitation is often based on a limited number of assessments. The development of SI after CI has rarely been detailed, although knowing the shape of the learning curve can potentially improve predictive modeling. Knowing the learning curve after CI could also aid in setting expectations about SI immediately after implantation, and the duration of rehabilitation. The current objectives were to construct learning curves to estimate baseline SI at 1 week (*B*), maximal SI after rehabilitation (*M*), and rehabilitation time (time to reach 80% of the learning effect; *t*[*M* − *B*]_80%_), and to subsequently deploy these outcomes for multiple-regression modeling to predict CI outcomes.

**Design:**

To assess rehabilitation after cochlear implantation, we retrospectively fitted learning curves using clinically available SI assessments from 533 postlingually deaf, unilaterally implanted adults. SI was assessed with consonant-vowel-consonant words (CVC) in quiet, with phoneme score as the outcome measure. Participants were followed for up to 4 years, with SI measurements collected at fixed intervals. SI was commonly assessed 1, 2, 4, and 8 weeks after device activation. *B*, *M*, and *t*(*M* − *B*)_80%_ were determined from the fitted learning curves. Predictive multiple-regression analyses were performed on these three outcome measures based on eight previously identified preoperative demographic and audiometric predictor variables: age at implantation, duration of severe-to-profound hearing loss, best-aided CVC phoneme score (in the free field), unaided ipsilateral and contralateral residual hearing and CVC phoneme scores (measured with headphones), and education type (regular or special education).

**Results:**

At 1 week after CI activation, raw phoneme scores had increased from 40% preoperatively (best-aided condition) to 51%, with further improvement to approximately 78% at 4 years. SI increased significantly until 1 year after activation and then plateaued. Fitted learning curves supported better estimates of these parameters, showing that average baseline SI at 1 week after CI activation was 51%, increasing to 85% after rehabilitation. The asymptotic score exceeded the raw average after 4 years because many cases had not yet plateaued. The median *t*(*M* − *B*)_80%_ was 1.5 months. Predictive modeling identified duration of hearing loss, age at implantation, best-aided CVC phoneme score, and education type as the most robust predictors for postoperative SI. Despite the statistically significant correlations, however, the combined predictive value was ~19% for *B*, 10% for *M*, and 2% for *t*(*M* − *B*)_80%_.

**Conclusions:**

This study is among the few to generate detailed learning curves after cochlear implantation. By including clinical SI measures in the earliest rehabilitation period, we report a median rehabilitation time with CI of 1.5 months. This implied rapid learning effect emphasizes the value of monitoring SI in the first few weeks after rehabilitation. According to multiple-regression analyses, the most commonly used preoperative variables correlated significantly with postoperative outcomes, but with limited predictive value for the clinic. By fitting learning curves through data reported in the literature, we show that the increase in SI during rehabilitation is an important predictor for *t*(*M* − *B*)_80%_.

## INTRODUCTION

A cochlear implant (CI) is currently the first-line treatment for severe-to-profound sensorineural hearing loss. Despite the considerable successes of CI treatment, speech perception outcomes vary substantially (Goudey et al. 2021). This variability calls for reliable methods of determining CI candidacy based on preoperative parameters. Much research effort has focused on identifying relevant factors, and several extensive multicenter investigations have firmly established the predictive value of several preoperative factors, including the duration of severe-to-profound hearing loss (DoHL) before implantation, age at implantation, etiology, and residual hearing (i.e., the average pure-tone audiometric threshold [PTA]) (Blamey et al. 1996, 2013; Lazard et al. 2012). In one recent meta-analysis (Zhao et al. 2020), however, age at implantation emerged as the only significant predictive factor among DoHL, age, PTA, and aided word recognition at the time of implantation.

The same group found that each identified predictor could explain less than 10% of the variance, calling the clinical relevance of these factors into question. Later multicenter studies confirmed that predictive models can explain approximately 12 to 20% of the variance in speech intelligibility (SI) outcomes of CI using linear methods (Goudey et al. 2021) and 18 to 22% with machine-learning approaches (Shafieibavani et al. 2021). One of the potential reasons for this low predictive power is the high degree of collinearity between the factors, which limits their cumulative explanatory value to approximately ≤20% of the observed variance (Midi et al. 2010; Lazard et al. 2012). For instance, age at implantation and PTA correlate because of age-related hearing loss (Yamasoba et al. 2013), whereas age and DoHL are collinear because DoHL can never outpace age.

This low predictive power limits the clinical value of these models (Velde et al. 2021). Generating more reliable outcome data is one possible way to better model CI outcomes. Many prediction models are based on a limited number of SI measurements, typically beginning 1 month after device activation and with follow-ups at progressively longer intervals, for example, at 3, 6, and 12 months (Ma et al. 2023). Despite a common assumption that post-implantation performance plateaus within a few months (Ma et al. 2023), some evidence indicates continued improvement long after this time (Colletti et al. 2011). Longer follow-up times thus could aid in better capturing SI trajectories after rehabilitation.

If serial SI measurements throughout the rehabilitation phase were applied in constructing learning curves (Holden et al. 2013), post-rehabilitation SI estimates might be more reliable than those based on the last available SI during follow-up. Because of their derivation from repeated SI assessments, fitted learning curves enhance precision by reducing susceptibility to test-retest variability. In addition, they are more accurate than single-point measurements after rehabilitation, given that performance may not have plateaued for all CI recipients at the time of a single assessment. Furthermore, learning curves from CI rehabilitation reveal additional features of CI outcomes, such as rehabilitation duration and SI levels achieved early after surgery. Understanding these variables is crucial for counseling purposes and managing expectations. Because the steepest post-implantation increase in SI typically occurs during the first month (Holden et al. 2013), measurements in this time window are critical for accurately fitting the learning curve. The typically sparse sampling over time and limited follow-up in many studies preclude such accurate fitting.

In this study, we used data from a large cohort of CI users who underwent SI measures at regular intervals until 4 years after implantation. We had access to multiple SI measures from the first weeks of rehabilitation, supporting the construction of reliable individual learning curves in the early phases. From each learning curve, we extracted three outcome measures: baseline SI at the start of rehabilitation, maximal SI after rehabilitation, and rehabilitation time (when 80% of improvement was reached between maximal and baseline, given as *t*[*M* − *B*]_80%_). Rehabilitation time and baseline SI have received little attention in past studies but can be essential outcomes for counseling.

As an illustration of the utility of the generated curves, we fitted them to postoperative data sets obtained from the literature (Zwolan et al. 2001; Adunka et al. 2008; Moberly et al. 2020; Grisel et al. 2022). We used the three outcome measures (baseline SI, maximal post-rehabilitation SI, *t*[*M* − *B*]_80%_) to build predictive multiple-regression models based on preoperative factors. Although Holden et al. (2013) used a similar approach and included postoperative predictors, we built our predictive models using only preoperative variables, which can be of more clinical value in predicting postoperative SI. To minimize redundancy in the model, we eliminated factors with excessive collinearity to better estimate the unique contribution of each variable (Dormann et al. 2013; Vatcheva et al. 2016). To contextualize our reported rehabilitation time, we fitted learning curves through data obtained from the literature.

## MATERIALS AND METHODS

### Study Design and Patients

This retrospective study included 533 postlingually bilateral deaf adults (300 biological females, 56%) who received a unilateral implant in our clinic. Adults are reimbursed for only one implant in the Netherlands as the standard of care. Additional comorbidities may be a reason to receive a second implant. As unilateral hearing loss is not an indication for CI, the number of unilateral deaf cases in the population was small, and the best contralateral ear had a PTA across 500 to 2000 Hz (PTA_500 to 2000_) of 33 dB HL. Only six cases had a PTA_500 to 2000_ better than 60 dB HL. “Postlingually” deaf is defined here after Van Dijkhuizen et al. (2011) as a loss of hearing after the age of 4 years. The population was implanted between January 1, 2000 and December 31, 2017, and included the first CI recipients treated in our center. Demographical, surgical, and audiological data were collected from electronic patient files. The few cases receiving bilateral CIs (n = 13) or who were reimplanted within the follow-up period (n = 6) were excluded from this study. Table 1 shows the demographic and audiometric profile of the 533 cases who met the inclusion criteria. From this cohort, 30 patients did not meet the learning curve fitting criteria (see later), so that 503 learning curves ultimately were obtained.

**TABLE 1.** Audiometric and demographic variables used for predictive modeling

	n	n (%)	Range	Median	IQR	Mean	SD
Age at implantation (yrs)	533	100	18–92	62	20	60	14
DoHL (yrs)	532	99.8	0–78	7	17	13	16
CVC_aided_ (%)	473	88.7	0–97	39	37	40	24
Education	481	90.2	n/a	n/a	n/a	n/a	n/a
CVC_ipsi_ (%)	532	99.8	0–87	15	39	21	22
CVC_contra_ (%)	530	99.4	0–97	37	52	35	28
PTA_ipsi_ (dB HL)	530	99.4	33–130	104	23	104	17
PTA_contra_ (dB HL)	532	99.8	18–130	95	26	95	20

Preoperative phoneme scores (in quiet) were occasionally high because CI candidacy depends primarily on speech-in-noise in our clinic and must be <50% at +5 dB SNR (Snel-Bongers et al. 2018).

CVC_ipsi_, unaided ipsilateral CVC phoneme score; DoHL, duration of hearing loss; Education, dichotomous parameter for regular (n = 429) or special education (n = 52); IQR, interquartile range; n/a, not applicable; n, number of cases where the predictor variable was available (total population, 533); PTA_500–2000_, average pure-tone audiometric threshold across 500–2000 Hz (dB HL); PTA_contra_, unaided contralateral PTA_500–2000_; PTA_ipsi_, unaided ipsilateral PTA_500–2000_ (dB HL).

### Implantation, Speech Processor Fitting, and Rehabilitation

Six surgeons performed the implantations using standard operating techniques. Implants were manufactured by Advanced Bionics, LLC (Valencia, CA, USA; n = 445, 83%), Cochlear Corp. (Sydney, Australia; n = 48, 9%), and MED-EL (Innsbruck, Austria; n = 40, 8%). Four to six weeks after implantation, a speech processor was fitted when the surgical site had sufficiently healed (designated as *t* = 0 in this study). Subsequently, patients were enrolled in an intensive 3-month training program for hearing rehabilitation, starting with two training sessions per day for the first 2 weeks and a decreasing training frequency after that (Frijns-van Putten et al. 2005).

### Demographic and Radiological Data

Before implantation, patients completed an intake questionnaire from which their education and DoHL were extracted, with DoHL defined as the number of years the participant could not use the phone. A subset of cases showed a “floor effect” on DoHL because they could still use the phone at the time of implantation. With this definition, however, DoHL correlated more robustly with SI during rehabilitation than did other measures of DoHL, such as self-reported age of onset of ‘severe hearing impairment’ in one or both ears. For this reason, inability to use a phone was retained as the criterion for self-reported DoHL. Education was included as a dichotomous, fixed variable for regular education (n = 429) or special education (n = 52). Only two levels of education were considered because the intake questionnaire elicited only limited details. Special education included schools for deaf or hearing-impaired students, for visually impaired students, and for those with learning disabilities. We note that attending schools for deaf students may have affected the exposure of patients to spoken language.

A total of 76 cases had cochlear abnormalities based on radiological outcomes. Cochlear abnormalities were not included as variables in the regression analyses because of the low incidence (14%) and the heterogeneity among the pathologies: 26 cases with cochlear ossification, 22 with otosclerosis, 8 with hypoplasia of the eighth cranial nerve, 5 with an enlarged vestibular aqueduct, 8 with severe cochlear malformation, and another 7 with suspected abnormalities that were not detailed.

Etiology was not considered in this study because the cause of hearing impairment was often unknown (Table 2). Age-related hearing loss is a prominent factor in our population, but not the leading indication for implantation, because most CI recipients had hearing losses that exceeded levels associated with age alone, implying involvement of other unknown causes. Cases with known etiology of hearing loss included familial history of hearing loss, confirmed genetic mutations, sudden deafness, and others (Table 2).

**TABLE 2.** Etiology of hearing loss in the study population

	n (Out of 533)	n (%)
Idiopathic progressive SNHL	198	37
Progressive SNHL with genetic and familial subtypes	114	21
Maternal *rubella*	10	2
Other congenital SNHL	57	11
Sudden deafness	42	8
Meningitis	29	5
Otosclerosis	22	4
Recurrent otitis	21	4
Ménière’s disease	19	4
Cholesteatoma	8	2
Ototoxicity	7	1
Trauma	4	1
Miscellaneous	2	< 1

Miscellaneous included HL induced by radiotherapy (n = 1) and HL due to an infection (n = 1). All 533 cases received their implant after acquiring full oral/aural language skills, including those diagnosed with congenital SNHL.

HL, hearing loss; SNHL, sensorineural hearing loss.

### Preoperative SI and Audiometric Testing

Speech recognition testing was performed according to Dutch Society of Audiology standards. Assessment was performed in a sound-attenuated audiometric booth with the Bosman speech corpus, consisting of monosyllabic consonant-vowel-consonant (CVC) words (Bosman & Smoorenburg 1995). The outcome measure was the CVC phoneme score. Each Dutch Society of Audiology list consisted of 12 CVC words, with the first excluded from scoring. The remaining 33 phonemes were used to determine SI. Each patient was asked to repeat verbally what they had heard. Phoneme scores have several advantages over the more conventional word score, including the available tokens in a given list. Phoneme scoring thus reduces outcome variability (or shortens test time) and the influence of the listener’s personal lexicon (reviewed in Billings et al. 2016). Because of these considerations, the phoneme score is the preferred measure for assessing SI in the Netherlands and Flanders. CVC phoneme scores are related to the more conventional word score by the following approximate relation (Bosman & Smoorenburg 1987; Bosman et al. 1992):


$$\begin{array}{l} W=P^{2.5} \end{array}$$ (1)

where W is the fractional word score and P is the fractional phoneme score.

**Fig. 1.**
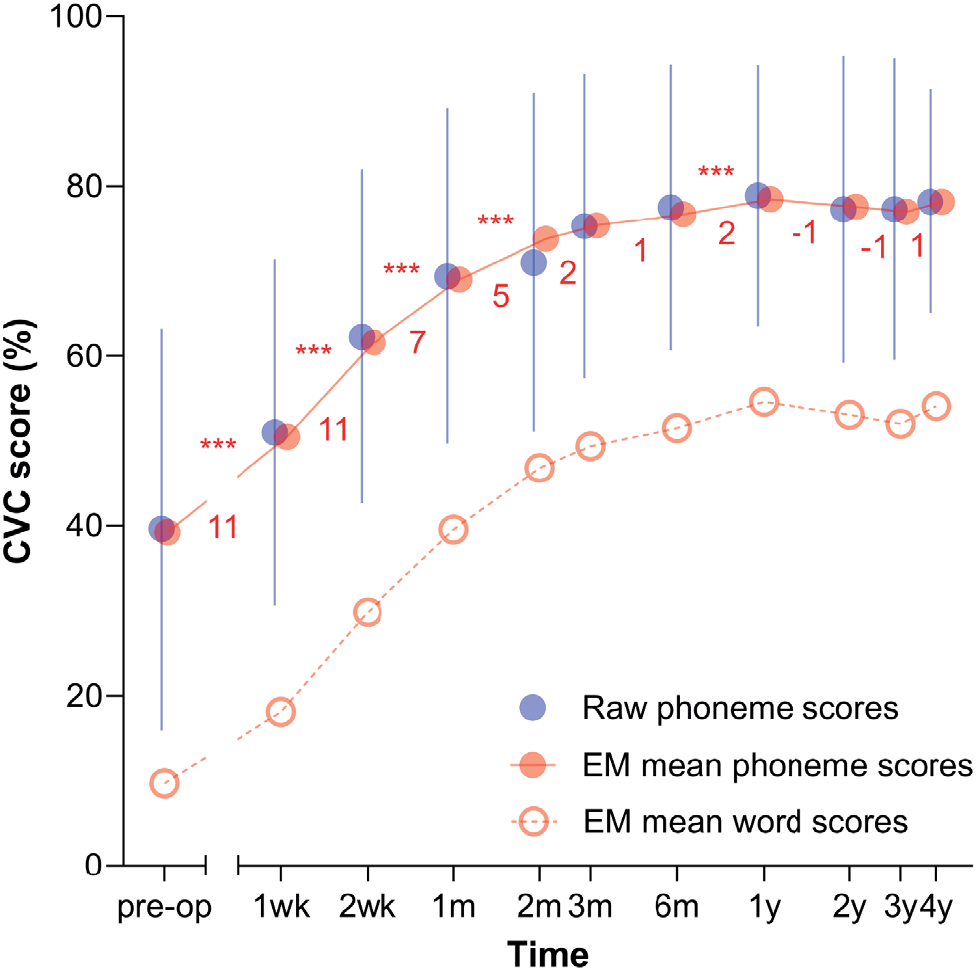
Preoperative and postoperative CVC phoneme scores during rehabilitation with a CI. Raw CVC phoneme scores (±SD, solid blue markers) and their corresponding EM means obtained from the LMM are shown (SEs ≤1%, solid red markers). The EM mean differences between consecutive time points obtained from the LMM are indicated (red numbers). ***p < 0.001. EM mean phoneme scores converted to word scores using Eq. (1) (see methods) are included (red open circles). CVC indicates consonant-vowel-consonant; EM, estimated marginal; LMM, linear mixed model.

Preoperative speech recognition SI was assessed in the free field and by using headphones (Table 3 and Fig. 1). The best-aided SI score was evaluated in the free field by allowing individuals to listen binaurally while using their assistive device(s) set to their preferred configurations, where applicable. A fitted hearing device that was deemed ineffective was refitted or replaced by a more effective device to ensure that implantation was the only option. CVC words were presented at 65 dB SPL in quiet using a loudspeaker situated directly in front of the patient at approximately 1 m distance. The final CVC phoneme score was obtained by averaging the scores across four lists (presented under the same conditions). Headphones were applied to assess the unaided, unilateral SI using standard procedures. Multiple presentation levels were applied to determine the maximal score using one list per level, with a maximum of 120 dB SPL. Audiometric testing was performed according to clinical protocol by slowly increasing the stimulus level until the listener indicated that the stimuli were uncomfortably loud, at which point the sound level was not increased.

**TABLE 3.** Descriptive statistics of preoperative and postoperative CVC phoneme scores

	Pre	1 wk	2 wks	1 mo	2 mos	3 mos	6 mos	1 yr	2 yrs	3 yrs	4 yrs
Score	40	51	62	69	71	75	77	79	77	77	78
SD	24	20	20	20	20	18	17	15	18	18	13
n	473	520	514	520	238	493	466	409	287	195	126
min	0	2	3	6	9	3	18	20	0	8	40
max	97	94	97	99	96	99	100	100	99	99	98
EMM	39.2	50.5	61.6	69.0	73.8	75.4	76.7	78.5	77.6	77.0	78.2
SE	0.9	0.9	0.9	0.9	0.9	0.8	0.8	0.7	0.8	0.9	0.8

EMM, SE, estimated marginal mean and SE from linear mixed model; m, month; min, max, minimum, maximum CVC score; n, number of cases with data at time point; Pre, preoperative; Score, mean CVC phoneme score (%); SD, SD (%); wk, week; y, year.

Preoperative pure-tone audiometry was performed to assess residual hearing using standard procedures. To convert the audiogram into a single predictor variable, the thresholds across 500, 1000, and 2000 Hz (PTA_500 to 2000_) were averaged, as recommended previously (Lazard et al. 2012). We chose this metric instead of the more commonly encountered PTA_500 to 4000_ because pure-tone thresholds at 4000 Hz were not measurable in many cases and thus lacked predictive value. Unmeasurable thresholds (>120 dB) were set at 130 dB HL to discern them from measurable thresholds ones of 120 dB HL.

### Speech Recognition Testing as a Measure of CI Outcome

Postoperative CI outcome was assessed via CVC phoneme scores obtained at regular intervals after 1 week, 2 weeks, 1 month, 2 months (dropped after 2011), 3 months, 6 months, and 1, 2, 3, and 4 years after activation of the CI. When all clinical follow-up appointment information was available, nine to ten test points could be included (Table 3 and Fig. 1). Testing was identical to the preoperative free-field method using the Bosman CVC words (Bosman & Smoorenburg 1995), yet only the CI was used. Contralateral hearing aids were removed, and the contralateral ear was plugged when residual hearing was substantial.

### Statistical Analysis of Raw SI Scores

SPSS for Windows v. 25 (IBM Corp. 2017, Armonk, NY, USA) was used for statistical analyses. The raw CVC phoneme scores of the 533 cases were analyzed with a linear mixed model and restricted maximum likelihood estimation to evaluate whether the SI differences between consecutive time points were statistically significant. Degrees of freedom were determined using Satterthwaite’s approximation (Satterthwaite 1946). The different time points were entered as a repeated fixed factor, and the CVC phoneme score was the outcome variable. Patient identifiers were included as a random intercept variable. An unstructured matrix was chosen based on the information criterion (Akaike or Bayesian) associated with commonly used variance/covariance matrix structures for repeated measures (e.g., compound symmetry, Toeplitz, diagonal, scaled identity). A scaled identity variance/covariance structure was chosen for the random variable because there was only one level. The estimated marginal (EM) mean phoneme scores at different time points were compared using post hoc multiple comparisons testing with Šidák’s correction.

### Learning Curve Fitting

**Fig. 2.**
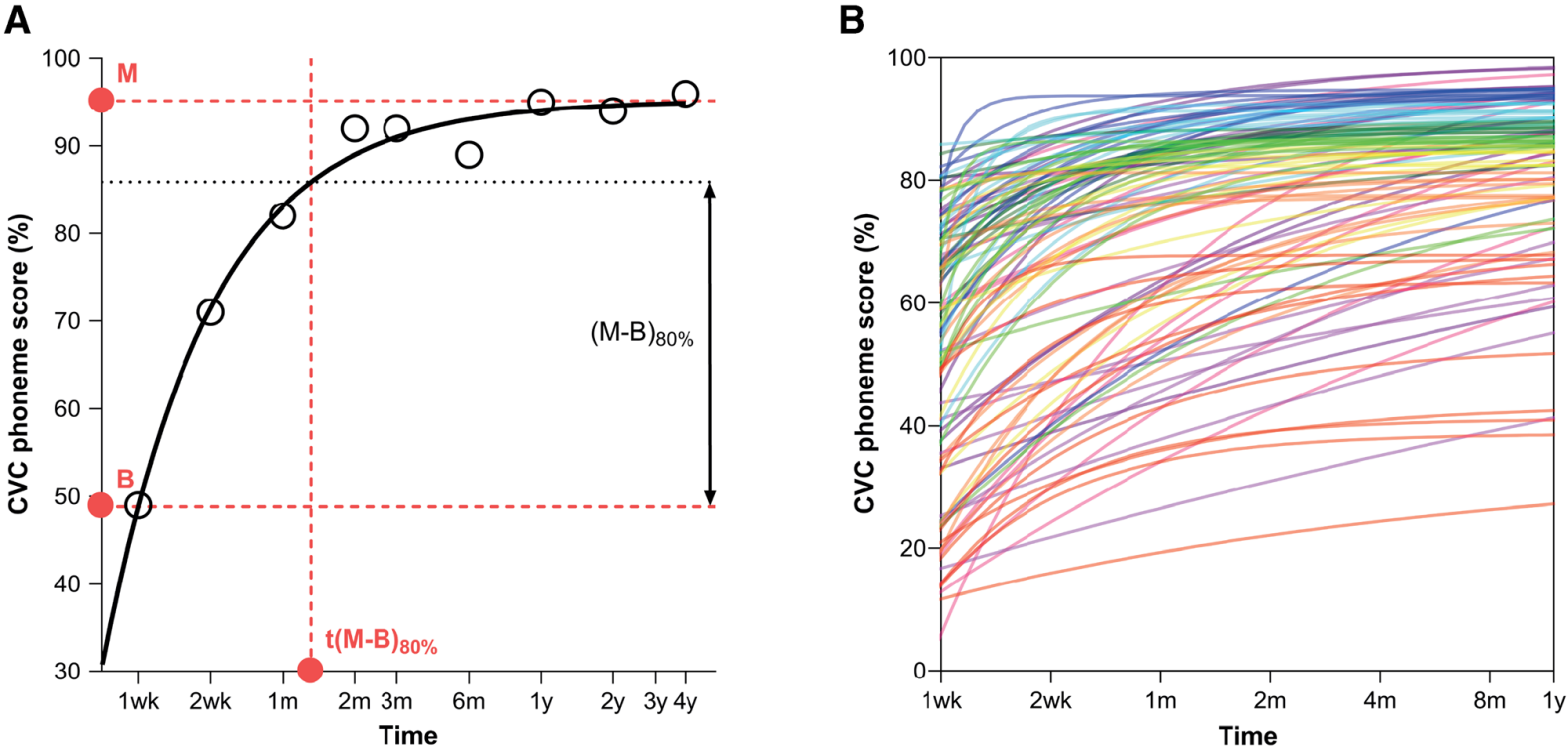
Sample fits of learning curves. A, Learning curve of one individual case. An exponential equation [Eq. (2), see methods] was fitted through the postoperative CVC phoneme scores (open circles) for each case using a logarithmic growth function (solid black line). From this learning curve, the speech intelligibility (SI) at 1 wk after CI activation (baseline, red *B* and dotted line), maximal SI after rehabilitation (horizontal asymptote, red *M* and dotted line), and the rehabilitation time (time to reach 80% of improvement *t*[*M* − *B* ]_80%_, vertical dotted red line at approximately 3 mos). *M* − *B*: improvement of CVC phoneme score during rehabilitation (black vertical arrow). B, 100 fits randomly selected from the data. Color signifies the deciles of *M*, with red lines illustrating the first decile (lowest CVC phoneme scores) and pink the upper decile (highest scores). CI indicates cochlear implant; CVC, consonant-vowel-consonant; SI, speech intelligibility.


Learning curves were fitted through the postoperative CVC phoneme scores for each case using an exponential growth function (Fig. 2A) with a nonlinear curve fitting procedure based on the least squares method in a MATLAB environment (R2021a, The MathWorks Inc., Natick, MA, USA; *lsqcurvefit* function):

$$\begin{array}{l} P=M–;\left(M–;a\right)\cdot e^{-\beta \cdot t} \end{array}$$ (2)

where P is the fractional CVC phoneme score, M is the maximal SI after rehabilitation (the horizontal asymptote of the curve), a is the intercept, β is the slope parameter, and t is the log_2_-converted time after device activation in months. The time axis was transformed because the CVC phoneme scores were obtained in progressively longer intervals. A more standard learning curve like the Weibull function (Gallistel et al. 2004) did not fit the data well because no apparent lower asymptote (i.e., “guess rate” γ in sigmoidal psychometric functions) was present (Wichmann & Hill 2001).

Of the 533 cases, 30 were excluded because they had <5 postoperative CVC phoneme scores available or the first assessment (1 week post-activation) was missing. The presence of the first measurement was deemed essential for reliable estimation of the slope in the steepest part of the learning curve. The fitted asymptote was capped at 100%.

Three main outcome measures were extracted from the curve fits: the interpolated baseline SI 1 week after activation (given as *B* in equations and figures), the maximal SI after rehabilitation (*M* in equations and figures), and the time point where SI reached 80% of the improvement (*t*[*M* − *B*]_80%_) (Fig. 2A). When baseline SI was equal to or greater than maximal SI, *t*(*M* − *B*)_80%_ could not be determined (n = 11). When maximal and baseline SI were the same, a horizontal linear fit was applied, so that they both equaled the average of the available CVC phoneme scores in these “non-learners.” We typically estimated *t*(*M* − *B*)_80%_ by interpolation, but extrapolation was sometimes necessary in case of incomplete follow-up or with continued improvement during the follow-up window. To prevent excessive measurement errors, an arbitrary cutoff criterion of 96 months (twice the follow-up time) was used, and *t*(*M* − *B*)_80%_ values >96 months were discarded (n = 75, including the 11 “non-learners”). Descriptive statistics of the fitted baseline SI, maximal post-rehabilitation SI, and *t*(*M* − *B*)_80%_ outcomes are shown in Table 4.

**TABLE 4.** Outcome measures obtained from fitting of learning curves

	Median	IQR	Mean	SD	Range	n (Out of 533)	n (%)
*M* (%)	89	16	85	15	0.1–100	503	94.4
*B* (%)	53	29	51	21	0.0–91	503	94.4
*t*(*M* − *B*)_80%_ (mos)	1.5	3.9	–	–	0.3–96	427	80.1

*t*(*M* − *B*)_80%_ was ^2^log transformed when regressed but presented in months here for clarity and skewed as a result; mean and SD are invalid consequently and not shown here.

*B*, baseline CVC phoneme score at 1 wk after surgery; IQR, interquartile range; *M*, maximal CVC phoneme score after rehabilitation; n, number of cases where the outcome measure was available (from the population of N = 533); *t*(*M* − *B*)_80%_, time to reach 80% of the improvement (months).

Missing values for dependent variables were interpolated (see later). Missing outcome measures were not interpolated because they often were missing based on exclusion criteria rather than randomly, and imputation could have led to unreliable data. Cook’s distances of the observed data never exceeded 1; therefore, we concluded that there were no influential outliers.

A previously used metric of rehabilitation time was the point at which 90% of the maximal SI was reached, that is, the “rise time” *t*(M)_90%_ (Holden et al. 2013). This metric was included as a secondary outcome measure and was typically reached slightly earlier than *t*(*M* − *B*)_80%_. In contrast to the “rise time,” *t*(*M* − *B*)_80%_ also can be adopted successfully for cases with SI improvement <10%, facilitating inclusion of cases with a high baseline SI metric (ceiling effects) and those with slight improvement overall after CI.

### Predictive Modeling Using Multiple Regression

For the multiple-regression analysis, the data were first preprocessed by using multiple imputation to substitute the missing data of the independent variables, under the assumption that data were missing at random (Schafer 1999; Sterne et al. 2009; Netten et al. 2017). The random number generator was set to Mersenne Twister, with a fixed starting value of 2,000,000 (the default). Five imputations were performed using fully conditional specification (Van Buuren 2007). Imputation constraints were set to the observed maximum and minimum variable values of each variable.

Multiple linear regression analyses were performed on the imputed data using SPSS. The independent variables included in the models depended on the outcome measure. To assess the collinearity of the independent variables, an initial multiple-regression analysis of the imputed data was performed, where every independent variable was cross-correlated with the others. The eight independent variables were as follows: DoHL, best-aided CVC phoneme score (CVC_aided_), age at implantation, education type, unaided ipsilateral and contralateral CVC phoneme scores (CVC_ipsi_ and CVC_contra_), and unaided ipsilateral and contralateral average pure-tone thresholds across 500 to 2000 Hz (PTA_ipsi_ and PTA_contra_).

Collinearity refers to the nonindependence of predictor variables. High collinearity inflates the variance of predictors and can lead to their incorrect identification as relevant predictors in a statistical model (Dormann et al. 2013). If the Pearson correlation between two variables exceeded 0.7, the one with the lower correlation with the outcome measure was discarded in the regression model (Dormann et al. 2013; Vatcheva et al. 2016). CVC_ipsi_ and PTA_ipsi_ exceeded the threshold for all outcome measures, as did the CVC_contra_ and PTA_contra_ pair. The unaided CVC phoneme scores correlated better with the outcomes in both cases. Consequently, PTA_ipsi_ and PTA_contra_ were omitted from any further analyses, and multiple-regression analyses were conducted with the remaining six predictors:


$$\begin{array}{lll} & Y=\left(a\cdot DoHL\right)+\left(b\cdot CVC_{aided}\right)+\left(c\cdot age\right) & +edu+\left(d\cdot CVC_{ipsi}\right)+\left(e\cdot CVC_{contra}\right)+F \end{array}$$ (3)

where Y is the outcome measure (baseline SI, maximal post-rehabilitation SI, or *t*[*M* − *B*]_80%_) and *F* is the regression constant in the model. For each outcome *Y*, is the predictive value of the independent variables is reported via the test statistic *t*, the chance level *p*, and the partial correlation squared (*r*_*p*_^2^), which signifies the percentage variance that the independent variable explains in the dependent variable after the linear effects of the other variables have been removed from both. Because regression was performed on a log_2_ time scale, the reported regression coefficients of *t*(*M* − *B*)_80%_ are on the same logarithmic scale unless explicitly stated otherwise. The resulting descriptive statistics in Table 4 (mean with SD and median with interquartile range [IQR]) are reported after conversion to months.

Analysis of the histograms of the residuals showed some skewing, which resulted at least in part from the capping of the data. The extracted outcome parameters of baseline SI and maximal post-rehabilitation SI were capped between 0% and 100% to respect the boundaries of speech recognition scores. For *t*(*M* − *B*)_80%_, a cap of 96 months was applied to prevent the occurrence of outliers (and substantially more skewing) from extrapolating too far outside the data range of 48 months. The non-normal distribution of residuals may have affected the reported regression coefficient estimates, *p* values, and SDs. Correlation metrics are robust against violations of normality, and the primary outcome statistic in this work was the coefficient of determination, reflecting how much of the observed variance was explained by the regression model (*r*^*2*^) or by its individual (partial) predictor variables (*r*_*p*_^2^).

### Contextual Analysis With Previously Published Data

As a final step of this work, we conducted a meta-analysis by fitting learning curves in the context of the few studies that report sufficiently detailed datasets for comparison (Zwolan et al. 2001; Adunka et al. 2008; Moberly et al. 2020; Grisel et al. 2022). For these analyses, we added the cases that had been excluded for the 96-month cutoff and also made comparisons of different rehabilitation outcome measures used here and in these earlier publications.

## RESULTS

### Analysis of the Raw SI Data With Linear Mixed Modeling

The raw averages of the preoperative and postoperative CVC phoneme scores are included in Table 3 and plotted in Figure 1 (solid blue markers). The EM means obtained from the linear mixed model (solid red markers in Figure 1) showed an increase of 39% in CVC phoneme scores in the first year after implantation. Subsequently, SI stabilized to an average of ~78%. During the first 2 months, the rate of SI increase was highest, resulting in a cumulative 34% improvement in the EM mean CVC phoneme score. Post hoc statistical significance testing showed that differences between the EM mean at consecutive time points were all significant in this period (*p* < 0.001). The SI increase in the intervals between 2 and 3 months (*p* = 0.061) and 3 and 6 months (*p* = 0.067) were not significant, yet between the 6-month and 1-year marks, there was another significant increase of 2% (*p* < 0.001). We note, however, that this increase is minor and falls within the expected test-retest variability of speech tests (Gelfand 1998).

### Learning Curve Fitting

A sample of 100 (approximately 20%) randomly selected fitted learning curves is shown in Figure 2B. The descriptive statistics of the extracted outcome variables of the entire population are shown in Table 4. CVC phoneme scores increased from 51% on average at baseline (1 week after CI activation) to 85% after rehabilitation. The median rehabilitation time was 1.5 months.

### Predictive Modeling

**Fig. 3.**
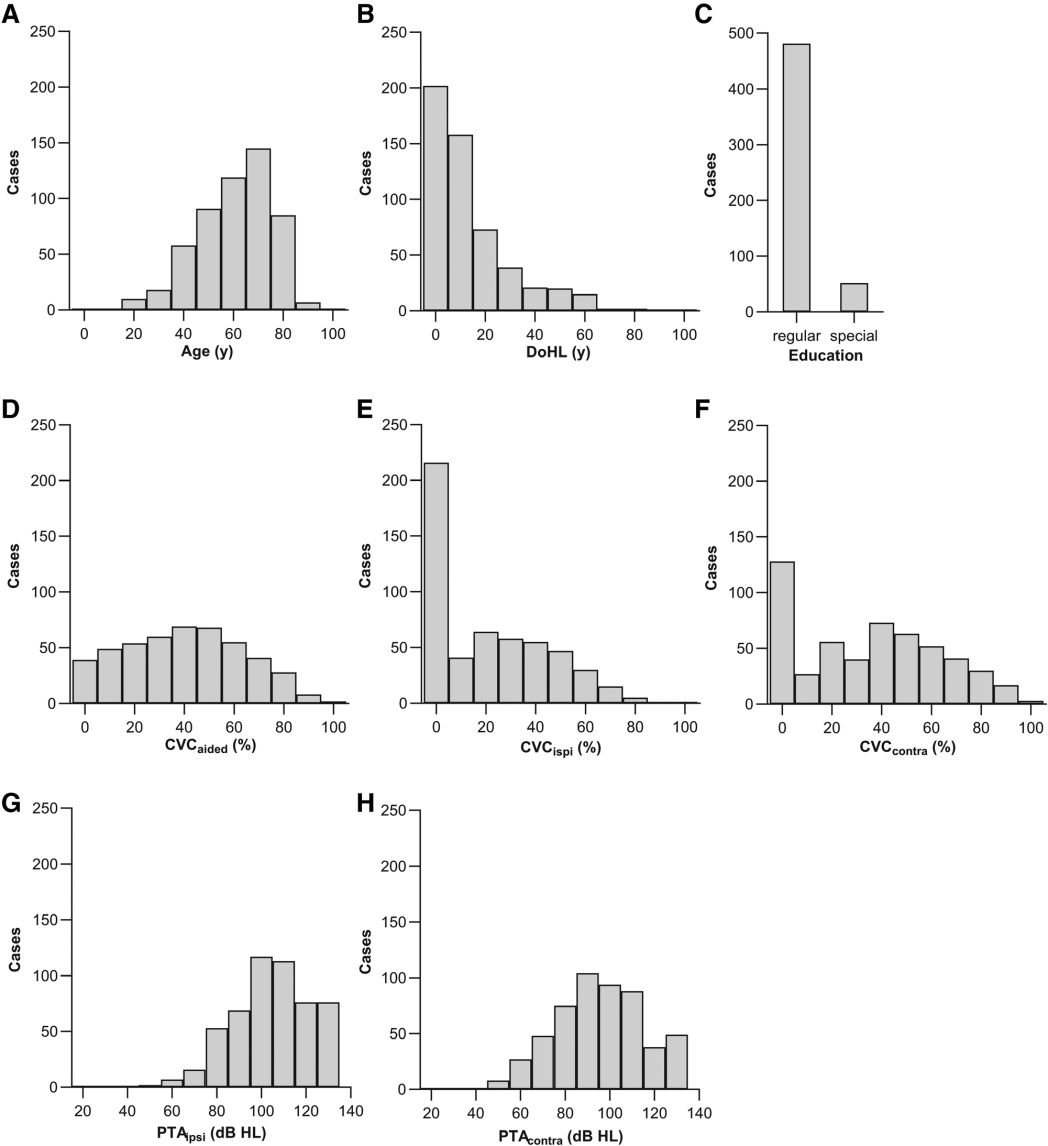
Histograms of the fixed variables used to predict CI outcomes with multiple-regression analyses. A, Age at implantation. B, Duration of severe-to-profound hearing loss. C, Education type, D, Best-aided preoperative CVC phoneme score. E and F, Unaided ipsilateral and contralateral phoneme scores. G and H, Unaided ipsilateral and contralateral average pure-tone thresholds across 500 to 2000 Hz. PTA_ipsi_ and PTA_contra_ were excluded from the regression models because of their collinearity with CVC_ipsi_ and CVC_contra_, respectively. CI indicates cochlear implant; CVC, consonant-vowel-consonant; PTA, pure-tone audiometric.

**Fig. 4.**
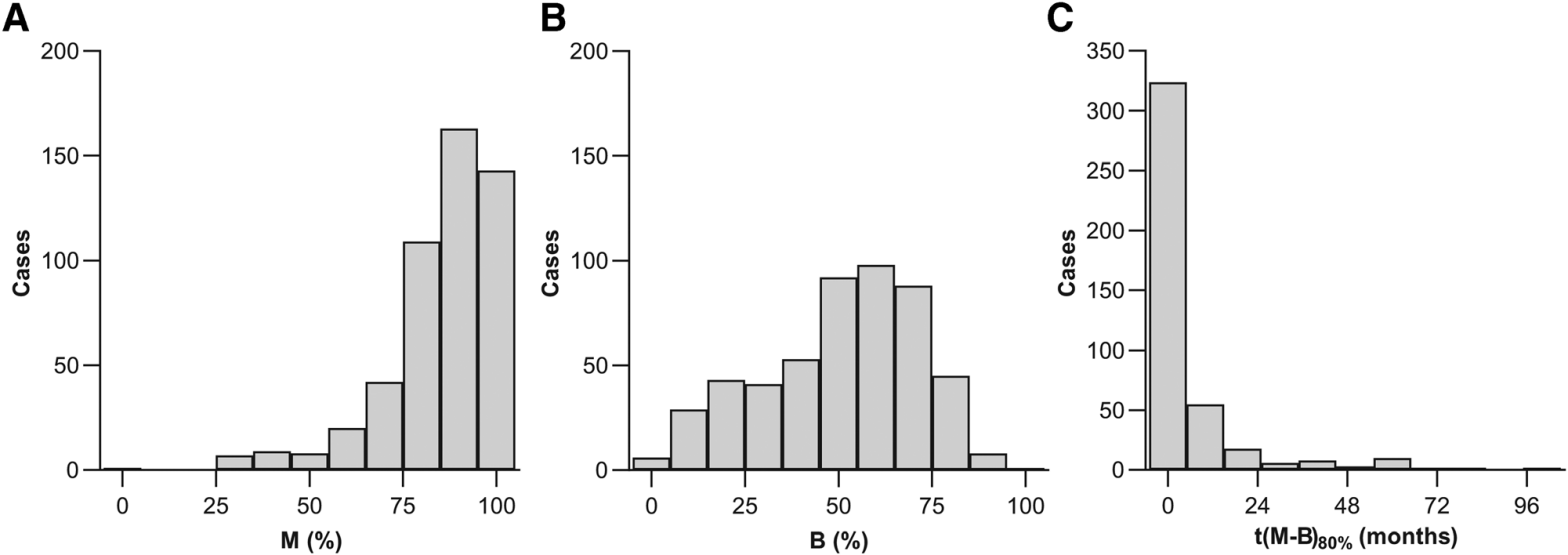
Histograms of the outcome variables used in the multiple-regression analyses. A, Maximal CVC phoneme score (*M*) after rehabilitation. B, Baseline CVC phoneme score (abbreviated as *B*) at the start of rehabilitation. C, rehabilitation time *t*(*M* − *B*)_80%_, that is, time to reach 80% of the improvement. CVC indicates consonant-vowel-consonant.

**Fig. 5.**
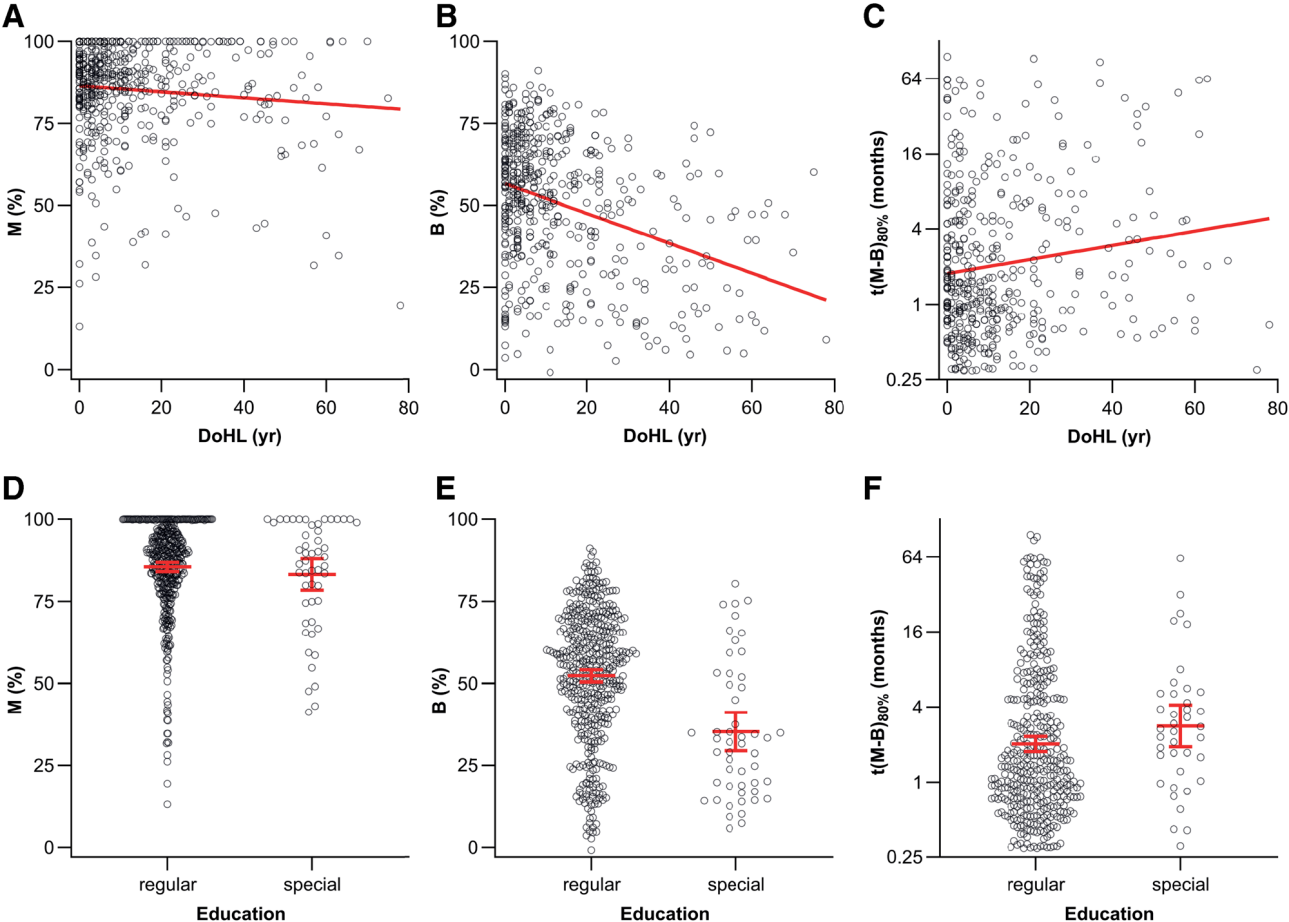
Sample correlations among the three outcome measures obtained from the learning curves and two of the six independent variables. A, Maximal SI after rehabilitation (*M*) as a function of the DoHL. B, Baseline SI at the start of rehabilitation (abbreviated as *B*) vs. DoHL. C, Rehabilitation time *t*(*M* − *B*)_80%_ vs. DoHL. D–F, Outcome measures *M*, *B*, and *t*(*M* − *B*)_80%_, respectively, as a function of education type. Red solid lines in A–C: bivariate correlations; red symbols in D–F: means with 95% confidence intervals. DoHL indicates duration of severe-to-profound hearing loss; SI, speech intelligibility.

Table 1 and Figure 3 show descriptive statistics of the original, non-imputed predictor variables. Table 4 and Figure 4 include descriptive statistics of the outcome measures. For illustrative purposes, we show the simple, bivariate correlation of the three outcome measures (baseline SI, maximal post-rehabilitation SI, *t*[*M* – B]_80%_) on DoHL in Figures 5A–C and the effect of the binary variable Education on the outcomes in Figures 5D–F.

#### Maximal CVC Phoneme Score After Rehabilitation

Multiple-regression analysis of the maximal CVC phoneme score using the imputed independent variables showed a significant predictive value of the model [*F*(6,494) = 9.740 to 11.480, *p* < 0.001]. The model’s adjusted *r*^2^ varied from 0.095 to 0.111 across the five multiple-imputed data sets, that is, the model explained 10 to 11% of the variance in SI after rehabilitation.

We identified a significant predictive effect of age at implantation (*r*_*p*_^2^ = 0.062, −0.3% [95% confidence interval = −0.4 to −0.2%] per year, *t* = −5.656, *p* < 0.001), best-aided CVC phoneme score (*r*_*p*_^2^ = 0.017, +0.1% [0.0 to 0.2%] per % CVC phoneme score, *t* = 2.671, *p* = 0.008), and education (*r*_*p*_^2^ = 0.010, −5.1% [−10 to −0.2%] if special education was attended, *t* = −2.061, *p* = 0.041). We found no significant correlations with DoHL (*r*_*p*_^2^ = 0.002, 0% [−0.1 to 0.0%] per year, *t* = −1.006, *p* = 0.315), ipsilateral unaided CVC phoneme score (*r*_*p*_^2^ = 0.004, 0.0% [0.0 to 0.1%] per %, *t* = 1.513, *p* = 0.130), or contralateral unaided CVC phoneme scores (*r*_*p*_^2^ = 0.000, −0.006% [−0.1 to 0.1%] per %, *t* = −0.184, *p* = 0.854).

#### Baseline CVC Phoneme Score at 1 Week After CI Activation

Regression of the baseline CVC phoneme score (interpolated at 1 week after CI activation) revealed a significant result [*F*(6,494) = 20.129 to 22.760, *p* < 0.001]. The model’s adjusted *r*^2^ varied from 0.186 to 0.191 and thus accounted for approximately 19% of the variance in the baseline data.

We found significant predictive effects for DoHL (*r*_*p*_^2^ = 0.061, −0.3% [−0.4 to 0.2%] per year, *t* = −5.658, *p* < 0.001), education (*r*_*p*_^2^ = 0.028, −11% [−17 to −5] for special education, *t* = −3.628, *p* < 0.001), and best-aided CVC phoneme score (*r*_*p*_^2^ = 0.022, +0.2% [0.1 to 0.3%] per % CVC phoneme score, *t* = 3.198, *p* = 0.001). There were no significant effects identified for age at implantation (*r*_*p*_^2^ = 0.001, 0% [−0.1 to 0.2%] per year, *t* = 0.678, *p* = 0.492), unaided ipsilateral CVC phoneme score (*r*_*p*_^2^ = 0.000, 0.0% [−0.1 to 0.1] per %, *t* = 0.376, *p* = 0.713), and contralateral CVC phoneme score (*r*_*p*_^2^ = 0.000, 0.0% [−0.1 to 0.1] per %, *t* = 0.377, *p* = 0.706).

#### Time to Reach 80% of Improvement

The multiple-regression model of the time to achieve 80% improvement (*t*[*M* − *B*]_80%_) showed a significant predictive effect [*F*(6,494) = 2.479 to 2.654, *p* = 0.023 to 0.015] with an adjusted *r*^2^ of 0.020 to 0.023 across the five imputed data sets, thus explaining ~2% of the variance in the data.

Regression was performed on log_2_-transformed values so that the resulting regression model coefficients do not translate directly to values in the time domain. Only DoHL had a significant predictive effect (*r*_*p*_^2^ = 0.014, +0.016 [0.003 to 0.029] per year, *t* = 2.428, *p* = 0.015). None of the other variables were significant: age at implantation (*r*_*p*_^2^ = 0.009, −0.014 [−0.027 to 0.000] per year, *t* = −1.957, *p* = 0.05), unaided ipsilateral CVC phoneme score (*r*_*p*_^2^ = 0.002, −0.004/% [−0.014 to 0005], *t* = −0.882, *p* = 0.378) per % CVC phoneme score, unaided contralateral CVC phoneme score (*r*_*p*_^2^ = 0.001, +0.003 [−0.007 to 0.013] per %, *t* = 0.663, *p* = 0.507), education (*r*_*p*_^2^ = 0.001, +0.22 [−0.4 to 0.9], *t* = 0.640, *p* = 0.522), and best-aided CVC phoneme score (*r*_*p*_^2^ = 0.001, −0.003/% [−0.014 to 0.009] per %, *t* = −0.46, *p* = 0.645).

### Contextual Analysis

We used the meta-analysis of Ma et al. (2023) as a reference for the rehabilitation time reported here. To contextualize our fitting of the learning curves and demonstrate their applicability, we included four studies from this meta-analysis (Zwolan et al. 2001; Adunka et al. 2008; Moberly et al. 2020; Grisel et al. 2022) in which SI data (i.e., CVC word scores in quiet) were reported in eight cohorts of CI users with sufficient detail [(see Eq. (2), methods]. None of these studies reported SI before 1 month postoperatively. We included the unaided, ipsilateral preoperative SI in the fits to cover the learning curve’s early (and steepest) phase. To facilitate a semi-logarithmic scale, an arbitrary time of 2 weeks was assigned to the preoperative SI. The same procedure was followed for our data using the EM mean word scores of Figure 1. The EM mean SI obtained at 1 and 2 weeks in our data were omitted from the fit to better compare it with those constructed from the literature.

**Fig. 6.**
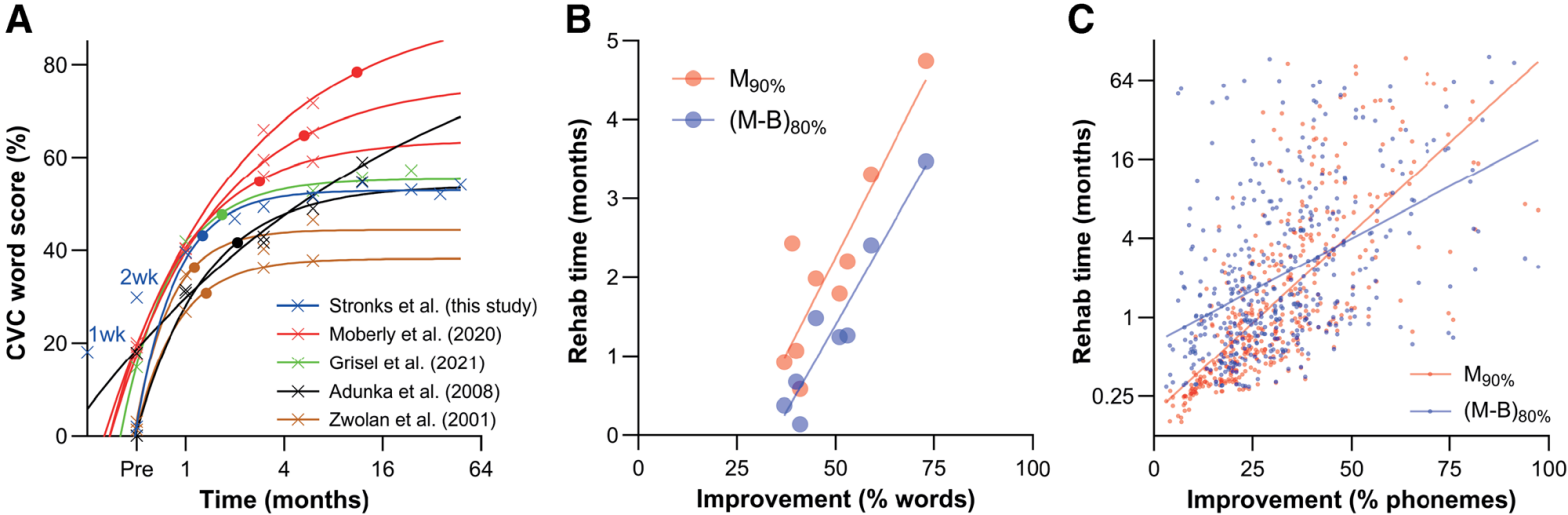
Dependence of rehabilitation time on SI improvement after cochlear implantation. A, Meta-analysis of longitudinal data from eight cohorts of CI users. CVC word scores are plotted against time after CI activation (cross markers). Learning curves [Eq. (2), see methods] are fitted through the data (solid lines), and rehabilitation times *t*(*M* − *B*)_80%_ determined generated (solid circles). The learning curve based on the EM estimated marginal mean CVC word scores [converted from phonemes using Eq. (1), see methods] from this paper are included (blue line), but word scores obtained at 1 and 2 wks after device activation were excluded from analysis (1 and 2 wks labels) to allow for better comparison. B, Correlations between two measures of rehabilitation time (*t*[*M* – *B*]_80%_ and “rise time” *M*_90%_) and the improvement during rehabilitation (*M* − *B*) using the data shown in A. Both measures correlated significantly (*r*^2^ = 0.9, *p* < 0.001 and *r*^2^ = 0.8, *p* < 0.01, respectively). C, Correlation between *t*(*M* − *B*)_80%_ and ‘rise time’ using the individually fitted learning curves generated in the current work. Correlations were again significant (*r*^2^ = 0.2, *p* < 0.001 and *r*^2^ = 0.5, *p* < 0.001, respectively). CVC indicates consonant-vowel-consonant; EM, estimated marginal.

The resulting fitted learning curves are shown in Figure 6A. The SI values obtained at 1 and 2 weeks in our data set (blue markers) were not used for curve fitting, and the steepness of the learning curve (blue line) was overestimated. As a consequence, *t*(*M* − *B*)_80%_ was shorter (1.2 months) than in our primary analysis (1.5 months). Visual examination revealed that *t*(*M* − *B*)_80%_ increased with SI after rehabilitation, indicating that the more people improved, the longer they needed to reach their asymptotic SI.

To investigate this observation further, we plotted *t*(*M* − *B*)_80%_ as a function of SI improvement *M* − *B* (Fig. 6B). Holden et al. (2013) investigated rehabilitation time using a similar approach but assessed the “rise time,” that is, the time to reach 90% of the final word score after rehabilitation. This metric inspired the *t*(*M* − *B*)_80%_ outcome (time to reach 80% of improvement) used here. Holden et al. reported a median “rise time” of 5.3 months in their population (n = 114), whereas our median value for *t*(*M* − *B*)_80%_ was 1.5 months (IQR = 3.9 months, n = 427). To contextualize for comparison here, we calculated our population’s median “rise time” as 0.9 months (IQR = 2.6, n = 427). When we included the 75 cases where *t*(*M* − *B*)_80%_ exceeded the 96-month criterion and assigned them an arbitrary value of 96 months, *t*(*M* − *B*)_80%_ (1.9 months; IQR: 13.7) and “rise time” (1.2 months, IQR: 10.1, n = 503) were slightly higher than our original values but still substantially smaller than the 5.3 months reported by Holden et al..

Figure 6B also includes the “rise time” (*M*_90%_) determined from the four studies cited in the 2023 meta-analysis of Ma et al. (2023) (Zwolan et al. 2001; Adunka et al. 2008; Moberly et al. 2020; Grisel et al. 2022). *t*(*M* − *B*)_80%_ could not be determined from Holden et al. (2013) because of a lack of numerical performance data in the report. As shown in the figure, both rehabilitation time measures *t*(*M* − *B*)_80%_ and “rise time” correlated significantly with improvement (*r*^2^ = 0.905, *p* < 0.001 and *r*^2^ = 0.791, *p* = 0.001, respectively). These results show that the magnitude of improvement can explain 91% of the variance in *t*(*M* − *B*)_80%_. We verified this conclusion in our data by plotting rehabilitation time against improvement in phoneme scores for each case individually (Fig. 6C). *t*(*M* − *B*)_80%_ and “rise time” correlated significantly with improvement (*r*^2^ = 0.216, *p* < 0.001 and *r*^2^ = 0.514, *p* < 0.001, respectively), showing that the magnitude of the SI increase can explain 22% of the variance in rehabilitation time.

## DISCUSSION

In this work, learning curves were obtained using data from a large cohort of unilaterally implanted CI users through retrospective analysis of SI measurements. The learning curves generated here allowed for reliable performance estimation after rehabilitation because measurements were available until 4 years after device activation. In addition, they allowed for extraction of a parameter for rehabilitation time and the SI at the start of rehabilitation for each CI recipient. We further report applying these learning curves for predictive modeling of CI outcomes.

### Learning Curves Enable Estimation of SI After Rehabilitation, Rehabilitation Time, and Baseline Score

Despite being an essential consideration for potential CI candidates, rehabilitation time just after device activation has received little attention. The rehabilitation time *t*(*M* − *B*)_80%_ of 1.5 months we report here shows that measurements in the first 2 months are required to assess the learning curve of implanted individuals. The increase in SI is steepest in this period. Holden et al. (2013) have published one of the few studies characterizing the learning curve shortly after implantation, investigating rehabilitation time using an approach similar to ours. Their “rise time” metric and the *t*(*M* − *B*)_80%_ outcome (time to reach 80% of improvement) used in the present study both accurately reflect rehabilitation time; however, the *t*(*M* − *B*)_80%_ metric allows for inclusion of cases where SI improves less than 10%, potentially preventing bias from exclusion of such cases. In our comparison of outcomes between Holden et al. and the present work, we found that our population’s median “rise time” was 0.9 months. When we added in the 75 cases for which *t*(*M* − *B*)_80%_ exceeded the 96-month criterion (assigning them an arbitrary value of 96 months), *t*(*M* − *B*)_80%_ was 1.9 months and “rise time” was 1.2 months, still substantially smaller than the 5.3 months reported by Holden et al.

We used the meta-analysis of Ma et al. (2023) as a reference for the rehabilitation time reported here. By pooling the preoperative and postoperative SI scores from 22 studies, these authors concluded that CI users improve significantly in the first 3 months but not after that. The current results refine this conclusion by showing that learning is steepest in the first 2 months, with 87% of the total improvement reached in this time window (34% improvement of a total of 39% at plateau). We captured this trend in our finding of a median *t*(*M* − *B*)_80%_ of 1.5 months. To contextualize our fitting of the learning curves, we included four studies from the 2023 meta-analysis (Zwolan et al. 2001; Adunka et al. 2008; Moberly et al. 2020; Grisel et al. 2022) in which sufficiently detailed SI data were reported. The fitted learning curves showed an overestimated steepness so that the *t*(*M* − *B*)_80%_ was 1.2 months as compared to the 1.5 months we identified in our original analysis. This difference emphasizes the relevance of taking SI measurements <1 month after implantation if learning curves are to be reliably predictive. The results further highlight that the more improvement there was in SI, the longer the time required to reach asymptote. In our analysis with earlier data and the current dataset, we also showed that both measures—that is, *t*(*M* − *B*)_80%_ and rise time—correlated significantly with SI improvement, and that the magnitude of this improvement explained 91% of the variance in *t*(*M* − *B*)_80%_. This finding was confirmed in our analysis of each case individually, indicating that the SI increase explained 22% of the variance in rehabilitation time.

Like rehabilitation time, the baseline score analyzed in the present work has not been described in many studies. We report an average baseline SI of 51% after device activation and a post-rehabilitation SI of 85%. The best-aided SI before implantation was 40%. As illustrated in Figure 6, a wide range of SI outcomes has been reported across various studies. Unlike SI after rehabilitation, however, baseline SI and rehabilitation time have received little attention in the literature even though both measures are essential for counseling because the rehabilitation process is intensive, often spanning multiple months. Providing realistic estimates of SI after implantation and the expected rehabilitation time can keep CI recipients motivated during rehabilitation, which is vital for maximizing CI outcomes (Frijns-van Putten et al. 2005).

### Predictive Modeling With Preoperative Factors

Together, the baseline SI, the expected SI after rehabilitation, and the rehabilitation time provide a complete picture of the implantation outcome. Developing a predictive model to estimate these parameters preoperatively for potential CI recipients is of clinical value. Using preoperative demographic and audiometric data, we have shown that DoHL, best-aided CVC phoneme score, age, and education type were significant predictors for these outcome measures. Yet, none of them had predictive power that was substantial enough to be of clinical value.

The preoperative variables that significantly correlated with CI outcomes differed across the three outcome measures. For maximal post-rehabilitation SI, the significant variables were age at implantation, best-aided CVC phoneme score, and education. Significantly correlated variables for baseline SI were DoHL, education, and best-aided CVC phoneme score, and for *t*(*M* − *B*)_80%_, DoHL was the only significant predictor. The multiple-regression models, which included all predictor variables, explained approximately 19% of the variance in baseline SI, 10% of the variance in maximal SI, and only 2% of the variance observed in *t*(*M* − *B*)_80%_. Across outcomes, even the most robust predictor variables (age at implantation and DoHL) did not explain more than 6% of the variance in the data. Overall, greater age and longer durations of deafness before implantation negatively impacted outcomes, whereas better best-aided preoperative hearing increased outcomes of CI. The regression estimates of special education attendance were associated with 5% lower outcomes for maximal post-rehabilitation SI and a 10% reduction in baseline SI. Unaided pure-tone thresholds and speech audiometric thresholds had negligible predictive value for any outcome measures and revealed high collinearity. We conclude that preoperative demographic and audiometric variables are not robust predictors of CI outcomes.

Previous studies investigating the predictive value of preoperative parameters have yielded similar findings. In their meta-analysis, Blamey et al. (1996) concluded that DoHL was the most robust predictor of speech perception outcomes, with more extended periods of deafness corresponding to poorer outcomes. Later studies have corroborated this finding (Van Dijk et al. 1999; Green et al. 2007; Budenz et al. 2011; Holden et al. 2013). A more recent meta-analysis identified DoHL as the only significant predictor (Zhao et al. 2020), with age at implantation, PTA, and aided word recognition showing only negligible predictive value. In an update on their original study, Blamey et al. (2013) reported that the duration of deafness still correlated with SI after implantation but accounted for a smaller part of the variability than they had previously reported. These authors contributed this shift to more lenient selection criteria for cochlear implantation over time, with people receiving their implant after a shorter period of hearing loss. The data used in the present work corroborate this trend, as DoHL was not a significant predictor for maximal post-rehabilitation SI and explained only 6% of the variance in baseline SI and 1% of the variance in *t*(*M* − *B*)_80%_. The phenomenon also emerged in the fact that more cases in later years had DoHL of zero, that is, people could still use the phone when they received their implant (data not shown). When the year of surgery was included in the current multiple-regression model, it showed considerable collinearity with the predictor variables, as anticipated. Collinearity was most prominent with preoperative aided and unaided phoneme scores (*r* = 0.4 to 0.5), reflecting the loosening of criteria for CI candidacy over time. However, the year of implantation did not significantly correlate with maximal post-rehabilitation SI, baseline SI, or *t*(*M* − *B*)_80%_.

Age at implantation correlated significantly only with maximal post-rehabilitation SI in our data and had a negative impact on the outcome. Other studies have generated mixed conclusions on this predictor, with some showing significant negative correlations with SI measures (Friedland et al. 2010; Blamey et al. 2013; Holden et al. 2013) and others showing none (Carlson et al. 2010; Budenz et al. 2011). One reason for this discrepancy may be collinearity of age at implantation with other predictors, notably DoHL. Older patients can have longer DoHL than those implanted at an early age. As a result, individual correlations will be attenuated when collinearly related factors are included in the model.

Most studies looking into the effects of cognitive SI conclude that it correlates significantly with SI outcomes (Heydebrand et al. 2007; Klop et al. 2007; Holden et al. 2013; Kaandorp et al. 2017; Moberly et al. 2017). Lazard et al. (2012) did not find an effect of education level on postoperative SI, but we found that special education is associated significantly and substantially with both baseline and maximal post-rehabilitation SI.

Holden et al. (2013) reported that DoHL and PTA in the pre-implanted CI ear had significant predictive value on the rehabilitation time *t*(M)_90%_. Earlier work in our department also identified DoHL as a significant predictor of the steepness of the learning curve (Briaire & Frijns 2008), and the current results corroborate these findings. However, we found no significant relationship between *t*(*M* − *B*)_80%_ and unaided ipsilateral or contralateral audiometric CVC phoneme scores, which both showed high collinearities with PTA_500 to 2000_. PTA_500 to 2000_ and speech recognition did not significantly correlate with any outcome measures. We hypothesize that the stringent implantation criteria in the Netherlands resulted in levels of residual hearing and unaided speech recognition that were too low to be of any postoperative predictive value.

Holden et al. (2013), among others (Lazard et al. 2012; Blamey et al. 2013), used preoperative and postoperative outcome measures, including time of CI use and insertion depth. The present study focused on preoperative factors to find relevant parameters for predicting postoperative SI. More important, we sought to critically assess the clinical value of a preoperative predictive model. We conclude that the factors evaluated here do not yield a viable model for preoperative prediction of CI outcomes. These factors comprised a set of eight potential audiometric and demographic predictors that were selected based on available literature. Together, they explained approximately 19% of the variance in CI baseline outcome, 10% of the asymptotic SI after rehabilitation, and 2% of the time to reach 80% of the learning effect. These predictive values are not robust enough to be of clinical value for determining CI candidacy or for counseling purposes.

Some weaknesses of this study deserve attention. Cases that resulted in unreliable learning-curve fits were excluded from analysis, including those where the curve was flipped, that is, the upper asymptote of the fitted learning was lower than the baseline (“non-learners”). This choice may have biased the data because those with the lowest phoneme scores were excluded from the analysis. Furthermore, stratification of cases could have made multiple regression more powerful. Holden et al. (2013) divided their study population into strata of SI after rehabilitation, using the maximal post-rehabilitation SI. We elected not to stratify in this way because this data processing is based on outcomes after rehabilitation; instead, we opted to use only preoperative parameters to build the regression models. In addition, collinearity between preoperative predictors was substantial. We removed instances where *r*^2^ between two fixed factors was >0.7, but collinearity between certain factors remained. As a result, the added value of including multiple predictors in the regression model was reduced. For instance, age, PTA_500 to 2000_, and unaided phoneme scores correlate because older people tend to have higher (worse) PTA_500 to 2000_ and lower scores with age-related hearing loss. Another limitation is that the population of CI users was obtained from a single center, which may have biased the outcomes and reduced the generalizability of the results. In addition, the etiologies in most cases were not known, and better and standardized reporting of etiology in patient files has been advised because of the potential predictive value it can offer (Goudey et al. 2021). Lastly, DoHL was obtained through self-reported questionnaires, and memory recall of medical events can be unreliable (Roberts et al. 1996).

## CONCLUSION

We report the generation of a detailed learning curve after cochlear implantation that incorporated data from the first few weeks of rehabilitation. The results show that rehabilitation time is primarily determined by how much SI will improve, explaining up to 90% of the variability in the population depending on the cohort and outcome measure under investigation. The implication is that CI recipients with low SI scores who improve a great deal after receiving their CI will need more time to reach their maximal SI level. However, predicting the rehabilitation time or other postoperative outcomes based on preoperative demographic and audiometric indicators for CI candidacy is challenging. Together, these preoperative factors explained approximately 19% of the variance of the initial SI with CI, 10% of the variance of the SI after rehabilitation, and just 2% of the variance of the time to reach 80% of the SI increase.

## ACKNOWLEDGMENTS

The authors thank Advanced Bionics for financial funds and Kees Haasnoot for his contributions to building the database.

## References

[R1] Adunka O. F. Buss E. Clark M. S. Pillsbury H. C. Buchman C. A. (2008). Effect of preoperative residual hearing on speech perception after cochlear implantation. Laryngoscope, 118, 2044–2049. 18813141 10.1097/MLG.0b013e3181820900

[R2] Billings C. J. Penman T. M. Ellis E. M. Baltzell L. S. McMillan G. P. (2016). Phoneme and word scoring in speech-in-noise audiometry. Am J Audiol, 25, 75–83. 26989823 10.1044/2016_AJA-15-0068 PMC4832875

[R3] Blamey P. Arndt P. Bergeron F. Bredberg G. Brimacombe J. Facer G. Larky J. Lindström B. Nedzelski J. Peterson A. Shipp D. Staller S. Whitford L. (1996). Factors affecting auditory performance of postlinguistically deaf adults using cochlear implants. Audiol Neurootol, 1, 293–306. 9390810 10.1159/000259212

[R4] Blamey P. Artieres F. Başkent D.. (2013). Factors affecting auditory performance of postlinguistically deaf adults using cochlear implants: An update with 2251 patients. Audiol Neurootol, 18, 36–47. 23095305 10.1159/000343189

[R5] Bosman A. Smoorenburg G. F. (1987). Differences in listening strategies between normal and hearing-impaired listeners. In Schouten M. E. H. (Ed.), The Psychophysics of Speech Perception (pp. 467–472). Springer Netherlands.

[R6] Bosman A. J., & Smoorenburg G. F. (1995). Intelligibility of Dutch CVC syllables and sentences for listeners with normal hearing and with three types of hearing impairment. Audiology, 34, 260–284. 8837785 10.3109/00206099509071918

[R7] Bosman A. J. Smoorenburg G. F. Bronkhorst A. W. (1992). Relations between phoneme scores and syllable scores for normal-hearing and hearing-impaired subjects. In Van Heuven V. J., & Pols L. C. W. (Eds.), The Auditory Processing of Speech. From Sounds to Words. Mouton de Gruyter.

[R8] Briaire J. J., & Frijns J. H. M. (2008). Chapter 7: The relative value of predictive factors of cochlear implant performance depends on follow-up time. In Cochlear Implants: From Model to Patients [Unpublished doctoral dissertation]. Leiden University.

[R9] Budenz C. L. Cosetti M. K. Coelho D. H. Birenbaum B. Babb J. Waltzman S. B. Roehm P. C. (2011). The effects of cochlear implantation on speech perception in older adults. J Am Geriatr Soc, 59, 446–453. 21361884 10.1111/j.1532-5415.2010.03310.x

[R10] Carlson M. L. Breen J. T. Gifford R. H. Driscoll C. L. Neff B. A. Beatty C. W. Peterson A. M. Olund A. P. (2010). Cochlear implantation in the octogenarian and nonagenarian. Otol Neurotol, 31, 1343–1349. 20729782 10.1097/MAO.0b013e3181edb69d

[R11] Colletti L. Mandalà M. Zoccante L. Shannon R. V. Colletti V. (2011). Infants versus older children fitted with cochlear implants: Performance over 10 years. Int J Pediatr Otorhinolaryngol, 75, 504–509. 21277638 10.1016/j.ijporl.2011.01.005

[R12] Dormann C. F. Elith J. Bacher S. Buchmann C. Carl G. Carré G. Marquéz J. R. G. Gruber B. Lafourcade B. Leitão P. J. Münkemüller T. McClean C. Osborne P. E. Reineking B. Schröder B. Skidmore A. K. Zurell D. Lautenbach S. (2013). Collinearity: A review of methods to deal with it and a simulation study evaluating their performance. Ecography, 36, 27–46.

[R13] Friedland D. R. Runge-Samuelson C. Baig H. Jensen J. (2010). Case-control analysis of cochlear implant performance in elderly patients. Arch Otolaryngol Head Neck Surg, 136, 432–438. 20479370 10.1001/archoto.2010.57

[R14] Frijns-van Putten A. A. M. E. Beers M. Snieder S. G. Frijns J.H.M. (2005). Hoortraining voor volwassen CI-dragers. Het cochleaire leermodel. Logopedie Foniatrie, 2, 50–59.

[R15] Gallistel C. R. Fairhurst S. Balsam P. (2004). The learning curve: Implications of a quantitative analysis. Proc Natl Acad Sci U S A, 101, 13124–13131. 15331782 10.1073/pnas.0404965101 PMC516535

[R16] Gelfand S. A. (1998). Optimizing the reliability of speech recognition scores. J Speech Lang Hear Res, 41, 1088–1102. 9771631 10.1044/jslhr.4105.1088

[R17] Goudey B. Plant K. Kiral I. Jimeno-Yepes A. Swan A. Gambhir M. Büchner A. Kludt E. Eikelboom R. H. Sucher C. Gifford R. H. Rottier R. Anjomshoa H. (2021). A multicenter analysis of factors associated with hearing outcome for 2,735 adults with cochlear implants. Trends Hear, 25, 23312165211037525. 34524944 10.1177/23312165211037525 PMC8450683

[R18] Green K. M. Bhatt Y. Mawman D. J. O’Driscoll M. P. Saeed S. R. Ramsden R. T. Green M. W. (2007). Predictors of audiological outcome following cochlear implantation in adults. Cochlear Implants Int, 8, 1–11. 17479968 10.1179/cim.2007.8.1.1

[R19] Grisel J. Miller S. Schafer E. C. (2022). A novel performance-based paradigm of care for cochlear implant follow-up. Laryngoscope, 132, S1–S10. 10.1002/lary.29614 34013978

[R20] Heydebrand G. Hale S. Potts L. Gotter B. Skinner M. (2007). Cognitive predictors of improvements in adults’ spoken word recognition six months after cochlear implant activation. Audiol Neurootol, 12, 254–264. 17406104 10.1159/000101473

[R21] Holden L. K. Finley C. C. Firszt J. B. Holden T. A. Brenner C. Potts L. G. Gotter B. D. Vanderhoof S. S. Mispagel K. Heydebrand G. Skinner M. W. (2013). Factors affecting open-set word recognition in adults with cochlear implants. Ear Hear, 34, 342–360. 23348845 10.1097/AUD.0b013e3182741aa7 PMC3636188

[R22] Kaandorp M. W. Smits C. Merkus P. Festen J. M. Goverts S. T. (2017). Lexical-access ability and cognitive predictors of speech recognition in noise in adult cochlear implant users. Trends Hear, 21, 2331216517743887. 29205095 10.1177/2331216517743887 PMC5721962

[R23] Klop W. M. Briaire J. J. Stiggelbout A. M. Frijns J. H. (2007). Cochlear implant outcomes and quality of life in adults with prelingual deafness. Laryngoscope, 117, 1982–1987. 17767086 10.1097/MLG.0b013e31812f56a6

[R24] Lazard D. S. Vincent C. Venail F.. (2012). Pre-, per- and postoperative factors affecting performance of postlinguistically deaf adults using cochlear implants: A new conceptual model over time. PLoS One, 7, e48739. 23152797 10.1371/journal.pone.0048739 PMC3494723

[R25] Ma C. Fried J. Nguyen S. A. Schvartz-Leyzac K. C. Camposeo E. L. Meyer T. A. Dubno J. R. McRackan T. R. (2023). Longitudinal speech recognition changes after cochlear implant: Systematic review and meta-analysis. Laryngoscope, 133, 1014–1024. 36004817 10.1002/lary.30354

[R26] Midi H. Sarkar S. K. Rana S. (2010). Collinearity diagnostics of binary logistic regression model. J Interdiscip Math, 13, 253–267.

[R27] Moberly A. C. Harris M. S. Boyce L. Nittrouer S. (2017). Speech recognition in adults with cochlear implants: The effects of working memory, phonological sensitivity, and aging. J Speech Lang Hear Res, 60, 1046–1061. 28384805 10.1044/2016_JSLHR-H-16-0119 PMC5548076

[R28] Moberly A. C. Vasil K. Baxter J. Klamer B. Kline D. Ray C. (2020). Comprehensive auditory rehabilitation in adults receiving cochlear implants: A pilot study. Laryngoscope Investig Otolaryngol, 5, 911–918. 10.1002/lio2.442 PMC7585234 33134539

[R29] Netten A. P. Dekker F. W. Rieffe C. Soede W. Briaire J. J. Frijns J. H. (2017). Missing data in the field of otorhinolaryngology and head & neck surgery: Need for improvement. Ear Hear, 38, 1–6. 27556528 10.1097/AUD.0000000000000346

[R30] Roberts R. O. Bergstralh E. J. Schmidt L. Jacobsen S. J. (1996). Comparison of self-reported and medical record health care utilization measures. J Clin Epidemiol, 49, 989–995. 8780606 10.1016/0895-4356(96)00143-6

[R31] Satterthwaite F. E. (1946). An approximate distribution of estimates of variance components. Biometrics, 2, 110–114. 20287815

[R32] Schafer J. L. (1999). Multiple imputation: A primer. Stat Methods Med Res, 8, 3–15. 10347857 10.1177/096228029900800102

[R33] Shafieibavani E. Goudey B. Kiral I. Zhong P. Jimeno-Yepes A. Swan A. Gambhir M. Buechner A. Kludt E. Eikelboom R. H. Sucher C. Gifford R. H. Rottier R. Plant K. Anjomshoa H. (2021). Predictive models for cochlear implant outcomes: Performance, generalizability, and the impact of cohort size. Trends Hear, 25, 23312165211066174. 34903103 10.1177/23312165211066174 PMC8764462

[R44] Snel-Bongers, J. Netten, A. P. Boermans, P. B. M. Rotteveel, L. J. C. Briaire, J. J. Frijns, J. H. M. (2018). Evidence-based inclusion criteria for cochlear implantation in patients with postlingual deafness. Ear Hear, 39, 1008–1014. 29642089 10.1097/AUD.0000000000000568

[R34] Sterne J. A. White I. R. Carlin J. B. Spratt M. Royston P. Kenward M. G. Wood A. M. Carpenter J. R. (2009). Multiple imputation for missing data in epidemiological and clinical research: Potential and pitfalls. BMJ, 338, b2393. 19564179 10.1136/bmj.b2393 PMC2714692

[R35] Van Buuren S. (2007). Multiple imputation of discrete and continuous data by fully conditional specification. Stat Methods Med Res, 16, 219–242. 17621469 10.1177/0962280206074463

[R36] Van Dijk J. E. van Olphen A. F. Langereis M. C. Mens L. H. Brokx J. P. Smoorenburg G. F. (1999). Predictors of cochlear implant performance. Audiology, 38, 109–116. 10206520 10.3109/00206099909073010

[R37] Van Dijkhuizen J. N. Beers M. Boermans P. P. Briaire J. J. Frijns J. H. (2011). Speech intelligibility as a predictor of cochlear implant outcome in prelingually deafened adults. Ear Hear, 32, 445–458. 21258238 10.1097/AUD.0b013e31820510b7

[R38] Vatcheva K. P. Lee M. McCormick J. B. Rahbar M. H. (2016). Multicollinearity in regression analyses conducted in epidemiologic studies. Epidemiology (Sunnyvale), 6, 227. 27274911 10.4172/2161-1165.1000227 PMC4888898

[R39] Velde H. M. Rademaker M. M. Damen J. Smit A. L. Stegeman I. (2021). Prediction models for clinical outcome after cochlear implantation: A systematic review. J Clin Epidemiol, 137, 182–194. 33892087 10.1016/j.jclinepi.2021.04.005

[R40] Wichmann F. A., & Hill N. J. (2001). The psychometric function: II. Bootstrap-based confidence intervals and sampling. Percept Psychophys, 63, 1314–1329. 11800459 10.3758/bf03194545

[R41] Yamasoba T. Lin F. R. Someya S. Kashio A. Sakamoto T. Kondo K. (2013). Current concepts in age-related hearing loss: Epidemiology and mechanistic pathways. Hear Res, 303, 30–38. 23422312 10.1016/j.heares.2013.01.021 PMC3723756

[R42] Zhao E. E. Dornhoffer J. R. Loftus C. Nguyen S. A. Meyer T. A. Dubno J. R. McRackan T. R. (2020). Association of patient-related factors with adult cochlear implant speech recognition outcomes: A meta-analysis. JAMA Otolaryngol Head Neck Surg, 146, 613–620. 32407461 10.1001/jamaoto.2020.0662 PMC7226297

[R43] Zwolan T. Kileny P. R. Smith S. Mills D. Koch D. Osberger M. J. (2001). Adult cochlear implant patient performance with evolving electrode technology. Otol Neurotol, 22, 844–849. 11698806 10.1097/00129492-200111000-00022

